# Nuclear fragile X mental retardation-interacting protein 1-mediated ribophagy protects T lymphocytes against apoptosis in sepsis

**DOI:** 10.1093/burnst/tkac055

**Published:** 2023-02-28

**Authors:** Peng-Yue Zhao, Ren-Qi Yao, Li-Yu Zheng, Yao Wu, Yu-Xuan Li, Ning Dong, Jing-Yan Li, Xiao-Hui Du, Yong-Ming Yao

**Affiliations:** Translational Medicine Research Center, Medical Innovation Research Division and Fourth Medical Center of the Chinese PLA General Hospital, Beijing 100853, China; Department of General Surgery, First Medical Center of the Chinese PLA General Hospital, Beijing 100853, China; Translational Medicine Research Center, Medical Innovation Research Division and Fourth Medical Center of the Chinese PLA General Hospital, Beijing 100853, China; Department of Burn Surgery, The First Affiliated Hospital of Naval Medical University, Shanghai 200433, China; Translational Medicine Research Center, Medical Innovation Research Division and Fourth Medical Center of the Chinese PLA General Hospital, Beijing 100853, China; Translational Medicine Research Center, Medical Innovation Research Division and Fourth Medical Center of the Chinese PLA General Hospital, Beijing 100853, China; Translational Medicine Research Center, Medical Innovation Research Division and Fourth Medical Center of the Chinese PLA General Hospital, Beijing 100853, China; Department of General Surgery, First Medical Center of the Chinese PLA General Hospital, Beijing 100853, China; Translational Medicine Research Center, Medical Innovation Research Division and Fourth Medical Center of the Chinese PLA General Hospital, Beijing 100853, China; Translational Medicine Research Center, Medical Innovation Research Division and Fourth Medical Center of the Chinese PLA General Hospital, Beijing 100853, China; Department of Emergency, The Second Hospital of Hebei Medical University, Shijiazhuang 050000, China; Department of General Surgery, First Medical Center of the Chinese PLA General Hospital, Beijing 100853, China; Translational Medicine Research Center, Medical Innovation Research Division and Fourth Medical Center of the Chinese PLA General Hospital, Beijing 100853, China

**Keywords:** NUFIP1, Ribophagy, Sepsis, Apoptosis, Immunosuppression, Autophagy, Mitophagy, Lymphocyte, Lipopolysaccharide

## Abstract

**Background:**

Ribophagy is a selective autophagic process that specifically degrades dysfunctional or superfluous ribosomes to maintain cellular homeostasis. Whether ribophagy can ameliorate the immunosuppression in sepsis similar to endoplasmic reticulum autophagy (ERphagy) and mitophagy remains unclear. This study was conducted to investigate the activity and regulation of ribophagy in sepsis and to further explore the potential mechanism underlying the involvement of ribophagy in T-lymphocyte apoptosis.

**Methods:**

The activity and regulation of nuclear fragile X mental retardation-interacting protein 1 (NUFIP1)-mediated ribophagy in T lymphocytes during sepsis were first investigated by western blotting, laser confocal microscopy and transmission electron microscopy. Then, we constructed lentivirally transfected cells and gene-defective mouse models to observe the impact of NUFIP1 deletion on T-lymphocyte apoptosis and finally explored the signaling pathway associated with T-cell mediated immune response following septic challenge.

**Results:**

Both cecal ligation and perforation-induced sepsis and lipopolysaccharide stimulation significantly induced the occurrence of ribophagy, which peaked at 24 h. When NUFIP1 was knocked down, T-lymphocyte apoptosis was noticeably increased. Conversely, the overexpression of NUFIP1 exerted a significant protective impact on T-lymphocyte apoptosis. Consistently, the apoptosis and immunosuppression of T lymphocytes and 1-week mortality rate in NUFIP1 gene-deficient mice were significantly increased compared with those in wild-type mice. In addition, the protective effect of NUFIP1-mediated ribophagy on T lymphocytes was identified to be closely related to the endoplasmic reticulum stress apoptosis pathway, and PERK–ATF4–CHOP signaling was obviously involved in downregulating T-lymphocyte apoptosis in the setting of sepsis.

**Conclusions:**

NUFIP1-mediated ribophagy can be significantly activated to alleviate T lymphocyte apoptosis through the PERK–ATF4–CHOP pathway in the context of sepsis. Thus, targeting NUFIP1-mediated ribophagy might be of importance in reversing the immunosuppression associated with septic complications.

Highlights

- The current research is a pioneering exploration of ribophagy in human diseases.
- The association between ribophagy and the endoplasmic reticulum stress apoptosis pathway might provide new ideas for multiple organelle interactions.
- NUFIP1-mediated ribophagy could act as a quality control method to maintain the functional homeostasis of the whole cell.
- NUFIP1 is expected to become a novel target for molecular intervention and drug therapy in septic patients.

## Background

Sepsis represents life-threatening organ dysfunction provoked by an uncontrollable host response to infection [1]. Recent epidemiological studies have shown an increase of >30 million septic patients globally each year, with a mortality rate of 30%, rendering sepsis a heavy health-care burden [2,3]. Patients with advanced sepsis die from various opportunistic infections, proving that sepsis-induced immunosuppression rather than the storm of inflammatory mediators was the main driving force behind this high morbidity and mortality [4].

Immunosuppression refers to a pathophysiological state in the late stage of sepsis, which mainly manifests as decreased proliferative ability and increased apoptosis of lymphocytes together with massive release of anti-inflammatory cytokines [5,6]. As a crucial cell type in adaptive immunity, T lymphocytes participate in various processes in immune responses. Increasing evidence has demonstrated that T-cell exhaustion appears to be one of the indispensable mechanisms underlying sepsis-induced immunosuppression; therefore, maintaining the number and function of T lymphocytes can indisputably and effectively alleviate sepsis-induced immunosuppression [7]. As crucial cells in the host immune response during sepsis, T lymphocytes are mainly composed of CD4^+^T cells and CD8^+^T cells, called helper T lymphocytes and effector T lymphocytes, respectively. Based on the literature and the previous work by our team, we mainly focused on CD4^+^T cells in the current study since CD4^+^T cells can effectively eliminate pathogenic microorganisms invading the human body and play an important role in sepsis-related immunity, while CD8^+^T cells play a key role in antitumor immunity [8–10]. Hotchkiss *et al*. first proposed that patients with sepsis had evident T-lymphocyte apoptosis in the thymus and spleen, which was directly associated with the severity of illness [11,12]. Preclinical studies showed that the apoptosis of thymic T lymphocytes was significantly elevated in mice subjected to cecal ligation and puncture (CLP) surgery and in a thermally injured model [13]. Correspondingly, alleviating T-lymphocyte apoptosis has been well-accepted as an efficient therapeutic strategy to improve the prognosis of sepsis [14]. Although numerous clinical drug trials targeting T-lymphocyte dysfunction are ongoing, including trails studying chloroquine, isotretinoin, triptolide, interleukin (IL)-2 and recombinant human growth hormone, the majority of these clinical trials have focused on anti-human immunodeficiency virus infection. Notably, clinical studies on IL-7, which is known to inhibit T-lymphocyte apoptosis and restore lymphocyte counts in septic patients, are also being carried out. Unfortunately, the two clinical studies on IL-7 were terminated due to drug supply or pharmacokinetic issues. Thus, there is an urgent need to explore novel biological therapeutic targets to reverse sepsis-induced immunosuppression by protecting T lymphocytes from apoptosis.

A body of evidence has confirmed that septic patients can have a variety of metabolic abnormalities, including increased peripheral glucose intake and demand for calories and protein, namely, a negative nitrogen balance characterized by high catabolism [15]. Then, the imbalance in protein homeostasis triggers a series of subsequent physiological and pathological alterations in cells, even leading to various diseases. Ribosomes, the main apparatus of protein translation, play an indispensable role in the maintenance of protein homeostasis. As a precision-assembled organelle, the ribosome activates a variety of ribosome quality control systems (RQCS), including ribophagy, to ensure the normal execution of its function [16]. Ribophagy refers to a kind of organelle-specific autophagy that can specifically degrade ribosomes and was first proposed by Kraft *et al*. in *Saccharomyces cerevisiae* in 2008 [17]. Then, in 2018, nuclear fragile X mental retardation-interacting protein 1 (NUFIP1) was identified as the specific receptor for starvation-induced ribophagy, providing an unambiguous intervention target for subsequent related studies [18]. It is well-known that moderate autophagy can sustain the intracellular balance and exert an unequivocal protective role in cells. Therefore, autophagy has been deemed an innovative target for sepsis due to its key roles in eliminating aberrant intracellular proteins and maintaining the functional homeostasis of multiple organelles [19,20]. For instance, previous studies have demonstrated that mitophagy and endoplasmic reticulum autophagy (ERphagy) are remarkably upregulated in sepsis and can significantly improve the deleterious outcomes of this condition [21,22]. However, whether sepsis can induce the upregulation of ribophagy and exert a protective influence similar to those of mitophagy and ERphagy remains unknown. Considering that apoptosis can lead to decreases in the number and function of T lymphocytes, which is one of the crucial mechanisms of sepsis-induced immunosuppression, and that autophagy, as a quality control mechanism of the body, can effectively alleviate the programmed death of T lymphocytes, we speculated that ribophagy, as a representative of selective autophagy, may protect T lymphocytes against apoptosis, thereby ameliorating sepsis-induced immunosuppression.

To verify the abovementioned hypothesis, in the present study, we first investigated the activity and regulation of NUFIP1-mediated ribophagy in T lymphocytes during sepsis. Then, lentivirally transfected cells and gene-defective mouse models were constructed to observe the effect of NUFIP1 deletion on the apoptosis of T lymphocytes. Finally, the underlying molecular mechanism was explored through corroborating animal experiments and inhibiting key molecules associated with the signaling pathway.

## Methods

### Animals

The wild-type (WT) mice utilized in the experiment were provided by the Institute of Laboratory Animals, Peking Union Medical College, Beijing, China. The mouse strain was C57BL/6 J, and the mice were all males that were between 6 and 8 weeks of age and weighed between 20 and 24 g. NUFIP1^+/−^ mice established on a C57BL/6 J background were provided by the Shanghai Model Organisms Center, Shanghai, China. To exclude the potential impact of off-target effect, we conducted the off-target analysis (supplementary data 1, see online supplementary material). All mice were housed in a specified pathogen free environment at 25°C with a light/dark cycle of 12 h and had free access to laboratory chow and water.

### Reagents

CD4 (L3T4) microbeads were provided by Miltenyi Biotec Company, Bergisch Gladbach, Germany. Lipopolysaccharide [*Escherichia coli* O127:B8, lipopolysaccharide (LPS)] was procured from Sigma-Aldrich, St Louis, MO, USA. Concanavalin A (Con A) was provided by Solarbio, Beijing, China. Annexin-V-phycoerythrin (PE) and 7-aminoactinomycin D (7-AAD) apoptosis detection kits were purchased from BD, San Diego, CA, USA. An anti-NUFIP1 antibody was obtained from Proteintech, Rosemont, IL, USA. Antibodies specific for protein kinase RNA-like ER kinase (PERK), phosphorylated (p)-PERK, C/EBP homologous protein (CHOP), Bcl-2, Bax, and cleaved (c)-Caspase3 were obtained from Cell Signaling Technology, Danvers, MA, USA. Antibodies specific for glucose-regulated protein 78 (GRP78), ribosomal protein L7 (RPL7) and RPL26 were obtained from Abcam, Cambridge, MA, USA. ER-Tracker red was obtained from Invitrogen, Carlsbad, CA, USA and Alexa Fluor 488-conjugated goat anti-rabbit IgG (H + L) and Alexa Fluor 594-conjugated goat anti-mouse IgG (H + L) were provided by Santa Cruz Biotechnology, Santa Cruz, CA, USA. Triton X-100 was purchased from Sigma, St. Louis, MO, USA and salubrinal (Sal) was obtained from Selleckchem, Houston, TX, USA. Hoechst 33258 was procured from APExBIO, Houston, TX, USA.

### Procedures for CLP

A sepsis model was established in mice by CLP. After 4% chloral hydrate and sodium pentobarbital (60 mg/kg) anesthesia was administered, the abdomen was first disinfected; the disinfected area was approximately a square with a side length of 2.5 cm. Then, a 1.0 cm long incision was made along the midline of the abdomen. The cecum was ligated ~1 cm from the distal end and pierced with a 21-gauge needle to squeeze out a small amount of feces to induce sepsis. Then, the cecum was placed back into the abdominal cavity, the peritoneum and skin were sutured layer by layer and 1 ml of 0.9% saline was subcutaneously injected into the neck. The successful establishment of the sepsis model was mainly determined by observing the symptoms of the mice and recording the 7-day mortality rate after CLP. The classic symptoms included lethargy, diarrhea and hair follicle erection, while the ideal 7-day mortality of CLP mice was 30–50%. Mice in the sham group underwent the same operation except that the ligation and puncture steps were not performed.

### Endotoxin model induced by LPS

LPS was dissolved in phosphate buffered saline (PBS). Male mice in the experimental group received an intraperitoneal injection of LPS at different concentrations (3, 5 and 10 mg/kg). In contrast, mice in the blank control group were injected intraperitoneally with PBS.

### Cell culture and processing

*Jurkat* cells were purchased from CTCC, Shanghai, China. Cell culture medium was prepared as RPMI 1640 medium containing 100 U/ml penicillin and 100 μl/ml streptomycin with 10% heat-inactivated fetal bovine serum. T lymphocytes were cultured in a 5% CO_2_, 37°C humidified incubator. In time-effect and dose-effect experiments, T lymphocytes were cultured for 6, 12, 24, 48 and 72 h under stimulation with 500 ng/ml LPS and compared with a PBS control group to assess the optimal time point; then, 0, 10, 50, 100, 500 and 1000 ng/ml LPS were administered for 24 h to identify the optimal dose. After the optimal time and dose were determined, cells were collected and subjected to subsequent experimental procedures, such as western blotting (WB), flow cytometry, laser scanning confocal microscopy (LSCM) and transmission electron microscopy (TEM).

### NUFIP1 lentivirus generation and transfection

Suspension cell-specific recombinant lentiviruses carrying an overexpression or knockdown (KD) construct for NUFIP1 were constructed by Jikai Biotechnology Company, Shanghai, China. To overexpress or knock down the expression of the NUFIP1 protein, the above constructed recombinant lentiviruses were introduced into *Jurkat* cells. Transfection of the recombinant lentiviruses was performed according to the manufacturer’s instructions. When a stably transfected cell line was formed, the transfection ratio of *Jurkat* cells was detected by flow cytometry, and protein expression in the NUFIP1-overexpression group and NUFIP1-KD group was verified by WB.

### Isolation of splenic CD4^+^ T lymphocytes

Under sterile conditions, mouse spleens were removed and rinsed twice with prechilled PBS. The mouse splenic tissue was carefully ground through a mesh screen and washed with PBS continuously. The cell suspension containing the ground spleen tissue was collected, added to a mouse organ mononuclear cell separation solution at a ratio of 1 : 1, and centrifuged at 3000 rpm for 15 min. The cells in the cloudy interface after centrifugation were collected as mouse mononuclear cells. Splenic CD4^+^ T lymphocytes were then isolated from the mouse mononuclear cells using a CD4^+^ T lymphocyte isolation kit with a positive selection MS column according to the manufacturer’s instructions. The isolated CD4^+^ cells were placed in cell culture medium and cultured in a 5% CO_2_, 37°C humidified incubator. Splenic CD4^+^ T lymphocytes were stimulated with Con A for 24 h prior to subsequent experiments.

### Isolation of murine peripheral blood mononuclear cells

Peripheral blood mononuclear cells (PBMCs) were isolated using a density gradient centrifugation-based PBMC isolation kit (TBDsciences, Tianjin, China) as recommended by the manufacturer. First, whole blood (about 800 μl per mouse) was obtained by retroorbital bleeding of mice using sodium heparin blood collection tubes and diluted with precooled PBS containing ethylene diamine tetraacetic acid (EDTA). Next, the washed cell suspensions were layered on top of a lymphoprep layer and centrifugated at 450 g for 30 min. Then, the cloudy layer in the middle containing PBMCs was collected and washed with PBS once before follow-up experiments were performed.

### TUNEL apoptosis assay

A one-step terminal-deoxynucleoitidyl transferase mediated nick end labeling (TUNEL) apoptosis assay kit (#C1089) was purchased from Beyotime Biotechnology, Shanghai, China. Pretreated T lymphocytes (cell count >2 × 10^6^) were collected and washed once with PBS. The cells were then fixed with 4% paraformaldehyde for 30 min and washed once with PBS. Next, the cells were resuspended in PBS containing 0.3% Triton X-100 and incubated for 5 min at room temperature. An appropriate amount of the TUNEL assay solution was prepared according to the instructions and the cells were washed twice with PBS. Then, 50 μl of TUNEL assay solution was added to the samples, which were incubated at 37°C for 60 min in the dark and washed twice with PBS. Finally, the samples were suspended in 250–500 μl of PBS and observed under a fluorescence microscope.

### Western blot analysis

Pretreated T lymphocytes (cell count >6 × 10^6^) were collected in EP tubes, and a mixture of RIPA lysis buffer, 1 : 50 protease inhibitor, and 1 : 100 phosphatase inhibitor was used to lyse the cells. The specific steps for protein extraction were as follows: incubation on ice and intermittent shaking for 30 min to obtain the cell homogenate, three freeze/thaw cycles in liquid nitrogen and then centrifugation at 4°C and 14 000 rpm for 30 min to obtain the supernatant. The supernatant was mixed with sodium dodecyl sulfate (SDS)-loading buffer at a ratio of 4 : 1 and finally boiled at 95°C for 5 min. The loaded amount of different protein samples was determined according to a standard curve and then the samples were loaded for SDS polyacrylamide gel electrophoresis (Pulilai, Beijing, China). The proteins in the sample gel were electrotransferred to a nitrocellulose membrane that was then blocked with milk or a blocking solution. Specific antibodies were incubated at a concentration of 1 : 1000 to determine the expression of NUFIP1 (12515–1-AP, 1 : 1000), RPL7 (ab72550, 1 : 1000), RPL26 (ab59567, 1 : 1000), Bcl-2 (#3498, 1 : 1000), Bax (#2772, 1 : 1000), GRP78 (ab21685, 1 : 1000), PERK (#3192S, 1 : 1000), p-PERK (#3179, 1 : 1000), activating transcription factor (ATF)4 (#11815, 1 : 1000), CHOP (#2895, 1 : 1000) and c-Caspase3 (#9664, 1 : 1000). An anti-β-actin mouse monoclonal antibody was used as the standard control for the internal reference gene. After shaking overnight at 4°C, a secondary antibody selected according to the species of the primary antibody was incubated at 1 : 5000 and the blot was finally exposed and developed on a western blot visualizer.

### LSCM

LSCM (Leica, Mannheim, Germany) was employed to observe the expression and aggregation of NUFIP1 in T lymphocytes, as well as its colocalization with a lysosomal marker [Lyso-Tracker, lysosomal associated membrane protein 2 (LAMP2)] and the autophagic marker light chain (LC)-3. After incubation at 37°C for ~30 min, the cells were collected in a flow tube and counted to ensure that at least 1 × 10^6^ cells were present. The cells were fixed with 4% paraformaldehyde at room temperature for 1 h and then 0.3% Triton X-100 was used to permeabilize the cells at room temperature for 20 min. Thereafter, the cells were blocked with 1% bovine serum albumin for 1 h at room temperature and then an anti-NUFIP1 antibody (1 : 200) was added for overnight staining in a 37°C incubator. The next day, the cells were incubated with secondary antibodies [fluorescein isothiocyanate (FITC) goat-anti-IgG and phycoerythrin (PE)-goat-anti-IgG] for 1 h at room temperature for staining. Afterward, anti-LAMP2 (1 : 1000) was added to the cells and incubated at room temperature for 1 h. Subsequently, the supernatant was discarded and the remaining liquid and cells were thoroughly mixed. Then, 20 μl of cells was dropped on a glass slide and mixed with 10 μl of 4′,6-diamino-nuclei 2-phenylindole (DAPI) to label nuclei. Finally, the cells were covered with a coverslip and observed with LSCM to assess cell colocalization. Notably, the cells needed to be washed with PBS three times before proceeding to the next step throughout the protocol.

### Transmission electron microscopy

Cells in the experimental and control groups were collected and counted to ensure that the cell count was not <1 × 10^7^. The cells were washed with PBS to remove the supernatant, fixed with 4% glutaraldehyde for 1 h at 4°C and washed three times with PBS. The specimens were fixed, sectioned, dehydrated in ethanol and then embedded in epoxy resin. Ultrathin electron microscope samples were prepared by sectioning with an ultramicro blade and stained with uranyl acetate and lead citrate. Finally, the cells were imaged under a transmission electron microscope (JEOL, Peabody, MA, USA) and the microscopic morphological changes in the organelles were observed.

### Detection of the lentiviral transfection rate

*Jurkat* cells were transfected with lentiviral-based small interfering RNA (siRNA) carrying a construct to KD or overexpress NUFIP1 genes. Since the transfected *Jurkat* cells carried green fluorescent protein, transfection could be observed by fluorescence microscopy and the transfection rate of the lentivirus could be accurately detected by flow cytometry.

### Flow cytometric analysis

Anti-CD3 [100 234 (Brilliant Violet 510)] and anti-CD4 [100 510 (FITC)] antibodies were purchased from BioLegend, San Diego, CA, USA. T cells from different groups were stained according to the manufacturer’s instructions and fixed with 1% paraformaldehyde for detection by flow cytometry. The apoptosis of T lymphocytes was measured by double staining with Annexin-V-PE and 7-AAD, and a single-negative tube and a double-negative tube were set up as controls. Different groups of cells were collected into flow tubes and counted to ensure that the cell count in each tube was not <2.5 × 10^5^, with three replicate tubes for each group. After washing the cells once with precooled PBS, the supernatant was discarded, 500 μl of binding buffer was added to resuspend the cells and the cells were washed by centrifugation at 1500 rpm for 5 min. Then, the supernatant was discarded and ~100 μl of the remaining cell solution was retained. Then, 5 μl of Annexin-V-PE and 5 μl of 7-AAD were added to each tube. After incubation for 15 min at room temperature in the dark, the cells were diluted with 200 μl of binding buffer and analyzed by flow cytometry using a FACScan (BD Biosciences, Mountain View, CA, USA) within 1 h.

### Measurement of cytokine levels

Whole blood (~800 μl per mouse) was obtained from mice by retroorbital bleeding using sodium heparin blood collection tubes and then centrifuged at 2500 rpm for 25 min, and the supernatant was considered mouse serum. Next, mouse serum samples from various groups were assayed with enzyme-linked immunosorbent assay kits (MyBioSource Inc., San Diego, CA, USA) to measure the levels of IL-2, IL-4, IL-10, interferon-γ (IFN-γ) and transforming growth factor-β1 (TGF-β1) according to the manufacturer’s protocols.

### Hoechst 33258 analysis

Different groups of cells were collected into flow tubes and counted to ensure that the count for each group of cells exceeded 2 × 10^6^ and the cells were washed three times with PBS. Then, the cells were fixed with 500 μl of 4% paraformaldehyde for 1 h at room temperature and washed three times with PBS again. The supernatant was discarded and the cells were resuspended in 1 ml of PBS. Then, 3 ml of 10 μg/ml Hoechst 33258 solution was added, mixed well and incubated at room temperature for 5 min. After washing with PBS, sections were prepared and cell apoptosis was observed under a fluorescence microscope.

### Histological examination

Dissected lungs, hearts, livers and kidneys from various groups of mice were fixed in 4% paraformaldehyde overnight at 4°C and embedded in paraffin blocks. Tissue cryosections (4–5 μm) were dewaxed and then stained with hematoxylin–eosin (HE) for histological assessment. Histological manifestations were observed and analyzed via microscopy (Nikon Instruments Co., Japan). Sections were independently assessed by two experienced histologists who were unaware of the groupings. The histological scores for organs were calculated based on a four-point scale [0 (none) to 3 (severe)] assigned to each criterion, and at least three microscopic areas were examined to score each specimen (supplementary data 2, see online supplementary material) [23].

### Experimental design and grouping

*In vitro* experiments, including those involving *Jurkat* cells and splenic CD4^+^ T lymphocytes, were performed with LPS to simulate a septic environment, while *in vivo* experiments were performed using both the classical CLP animal model and the endotoxin model. To investigate the occurrence of ribophagy in sepsis, *Jurkat* cells and splenic CD4^+^ T lymphocytes were each divided into two groups: control and LPS groups (*n* = 3). *In vivo* experiments were divided into a sham group and a CLP group (*n* = 10). To investigate the effect of ribophagy on T-lymphocyte apoptosis in sepsis, *Jurkat* cells were divided into control, negative 1, KD, negative 2 and overexpression groups (*n* = 3). Splenic CD4^+^ T lymphocytes were divided into WT-control, WT-LPS, KD-control and KD-LPS groups (*n* = 5). To assess the impacts of ribophagy on the 1-week mortality of septic mice, animals were divided into four groups: WT-sham, WT-CLP, KD-sham and KD-CLP groups or WT-saline, WT-LPS, KD-saline and KD-LPS groups (*n* = 10). To explore the regulatory mechanism by which ribophagy limits the apoptosis of T lymphocytes, *Jurkat* cells were divided into six groups: control, control + LPS, control + LPS + Sal, KD, KD + LPS and KD + LPS + Sal groups (*n* = 3). Similarly, splenic CD4^+^ T lymphocytes were divided into six groups, namely, WT-control, WT-LPS, WT-LPS + Sal, KD-control, KD-LPS and KD-LPS + Sal groups (*n* = 3). To further evaluate the influence of Sal treatment on the 1-week survival rate of septic mice, animals were divided into six groups: WT-sham, WT-CLP, WT-CLP + Sal, KD-sham, KD-CLP and KD-CLP + Sal groups (*n* = 10).

### Statistical analysis

All statistical analyses were performed using IBM SPSS Statistics 24 and GraphPad Prism 8 software. Continuous data are presented as the means and standard deviations and categorical/ranked data are presented as counts and percentages where applicable. An unpaired Student’s t test and one-way analysis of variance (ANOVA) were used to determine the statistical significance when two groups and more than three groups, respectively, were compared. The data of flow cytometry were analyzed by FlowJo Version 10.0 software. Mouse survival curves were drawn using GraphPad Prism 8 and the difference in the survival rate was tested by the log-rank test. A *p* value < 0.05 was considered statistically significant.

## Results

### Apoptosis of T lymphocytes in sepsis

CD4^+^ T lymphocytes harvested from the mouse spleen were stimulated with Con A to induce proliferation, treated with 500 ng/ml LPS and collected at various time points for subsequent TUNEL apoptosis assay and western blot analyses. As shown in supplementary data 3 a, b (see online supplementary material), the apoptosis of splenic CD4^+^T lymphocytes and *Jurkat* cells showed a trend of first increasing and then decreasing, peaking 24 h after LPS stimulation. Moreover, LPS induced Caspase-3 activation (cleaved), upregulated Bax protein expression and suppressed Bcl-2 expression in splenic CD4^+^ T lymphocytes and *Jurkat* cells in a time-dependent manner. The differences identified by comparison with the control group were most significant at 24 h after stimulation (supplementary data 3 c–f, see online supplementary material). We compared the expression levels of c-Caspase3, Bax and Bcl-2 between the CLP and sham groups. Consistently, the WB results showed that CLP *per se* significantly induced T-lymphocyte apoptosis at 24 h after the operation, and quantitative densitometric analysis revealed a significant difference (supplementary data 3 g, h, see online supplementary material).

### Alterations of ribophagy in T lymphocytes during sepsis

**Figure 1:**
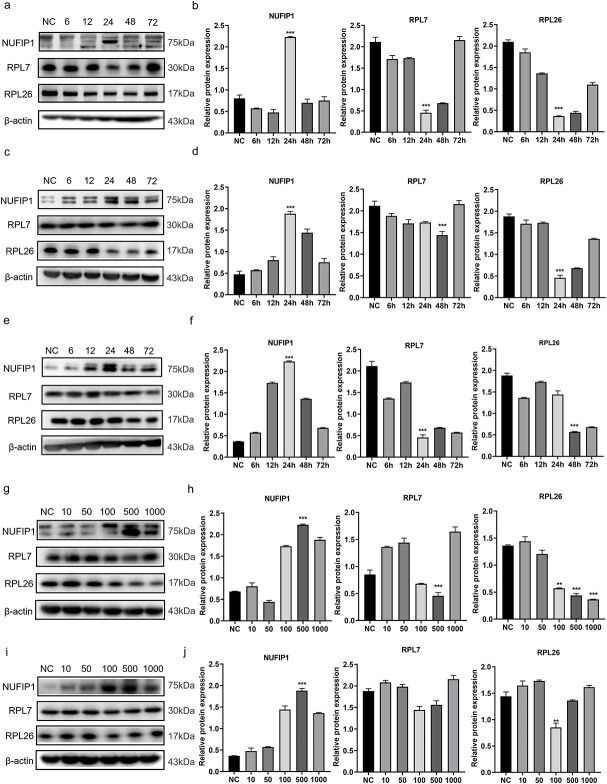
Time-effect and dose-effect responses of ribophagy in sepsis. (**a, b**) The time-effect response of splenic CD4^+^ T lymphocytes *in vitro*. After LPS stimulation for 6, 12, 24, 48 and 72 h, the protein levels of NUFIP-1 in splenic CD4^+^ T lymphocytes showed a trend of an initial increase and then decrease, and they peaked at 24 h. However, RPL7 and RPL26 expression exhibited a trend of an initial decrease and then increase, with the lowest value observed at 24 h. (**c, d**) Time-effect response of *Jurkat* cells. (**e, f**) The *in vivo* time-effect response. (**g**–**j**) The *in vitro* dose-effect experiment. After stimulating splenic CD4^+^ T lymphocytes or *Jurkat* cells with 10, 50, 100, 500 and 1000 ng/ml LPS for 24 h, expression of the NUFIP1 protein was upregulated in the stimulated group compared to the blank control group, while that of RPL7 and RPL26 was downregulated. Among the groups, the 500 ng/ml LPS stimulation group exhibited the most significant increase or decrease. One-way ANOVA was applied to test the statistical significance. Data are expressed as means ± SEM; ^*^^*^p < 0.01, ^*^^*^^*^p < 0.001. *NC* normal control group, *NUFIP1* nuclear fragile X mental retardation-interacting protein 1, *RPL7* ribosomal protein L7, *RPL26* ribosomal protein L26, *LPS* lipopolysaccharide, *ANOVA* analysis of variance, *SEM* standard error of mean

To investigate the activity and regulation of ribophagy in T lymphocytes during sepsis, we stimulated splenic CD4^+^ T lymphocytes and *Jurkat* cells with LPS at 500 ng/ml for 6, 12, 24, 48 and 72 h. WB results showed that with the prolongation of LPS stimulation, the protein expression of NUFIP1 showed a trend of initially increasing and then decreasing, peaking at 24 h (Figure 1a, c). As shown in Figure 1b, d, quantitative densitometric analysis of the protein level in the 24 h group showed that the level was significantly higher in the experimental group than in the control group, and the difference was statistically significant (*p* < 0.001). Furthermore, the protein expression levels of RPL7 and RPL26, which are essential components of the ribosome large subunit, showed trends opposite that of NUFIP1 (Figure 1a–d). Subsequently, we validated the experimental results *in vivo* by establishing sham and CLP animal models. WB results and quantitative densitometric analysis of protein levels indicated that the NUFIP1 level increased over time, peaked at 24 h and then gradually declined. In contrast, the RPL7 and RPL26 levels showed completely opposite trends (Figure 1e, f). When the optimal time point was determined, a dose-escalation experiment was conducted to explore the optimal concentration of LPS stimulation. Various concentrations of LPS (10, 50, 100, 500 and 1000 ng/ml) were utilized to stimulate splenic CD4^+^ T lymphocytes and *Jurkat* cells for 24 h. As shown in Figure 1g–j, the expression level of NUFIP1 gradually increased with increasing LPS concentration, while the RPL7 and RPL26 levels accordingly decreased in the LPS-stimulated groups compared with the control group, and the difference was most significant in the 500 ng/ml stimulated group (*p* < 0.01).

**Figure 2:**
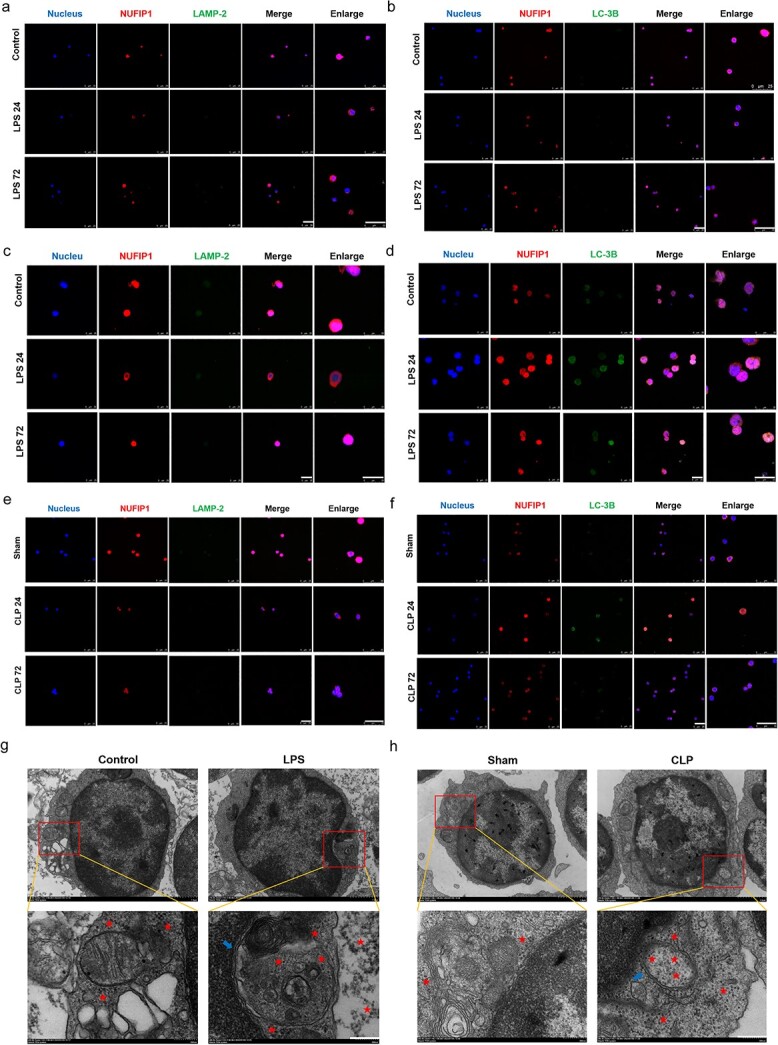
Ribophagy was significantly activated in sepsis. (**a, b**) LSCM examination indicated that the NUFIP-1 protein was scattered in both the cytoplasm and nucleus but mainly located in the nucleus. The expression of NUFIP1 was significantly increased in splenic CD4^+^ T lymphocytes after 500 ng/ml LPS stimulation for 24 h compared with control treatment, and NUFIP1 shuttling from the nucleus to the cytoplasm was observed. In addition, the colocalization of NUFIP1 with LC-3B and LAMP-2 was substantially intensified in the LPS-stimulated group in comparison to the control group. When the LPS stimulation time was increased to 72 h, the above changes gradually decreased, becoming close to the level observed before LPS stimulation (scale bar: 25 μm). (**c, d**) The expression of NUFIP1 and the fusion of NUFIP1 with LC-3B and LAMP-2 in *Jurkat* cells after 500 ng/ml LPS stimulation for 24 and 72 h compared with control groups (scale bar: 25 μm). (**e, f**) The expression of NUFIP1 and the fusion of NUFIP1 with LC-3B and LAMP-2 in splenic CD4^+^ T lymphocytes after CLP for 24 and 72 h compared with that in the sham group (scale bar: 25 μm)*.* (**g, h**) TEM showed that compared with the control or sham group, the LPS or CLP group showed an obviously swollen ER in splenic CD4^+^ T lymphocytes and autophagosomes containing a large number of ribosomes to be degraded [scale bars: 1 μm (top row) and 200 nm (bottom row)]. The red stars indicate the ribosomes and the blue arrows represent autophagosomes. *LSCM* laser scanning confocal microscopy, *TEM* transmission electron microscopy, *NUFIP1* nuclear fragile X mental retardation-interacting protein 1, *LAMP2* lysosomal associated membrane protein 2, *LC3B* light chain 3B, *LPS* lipopolysaccharide, *CLP* cecal ligation and puncture, *ER* endoplasmic reticulum

**Figure 3:**
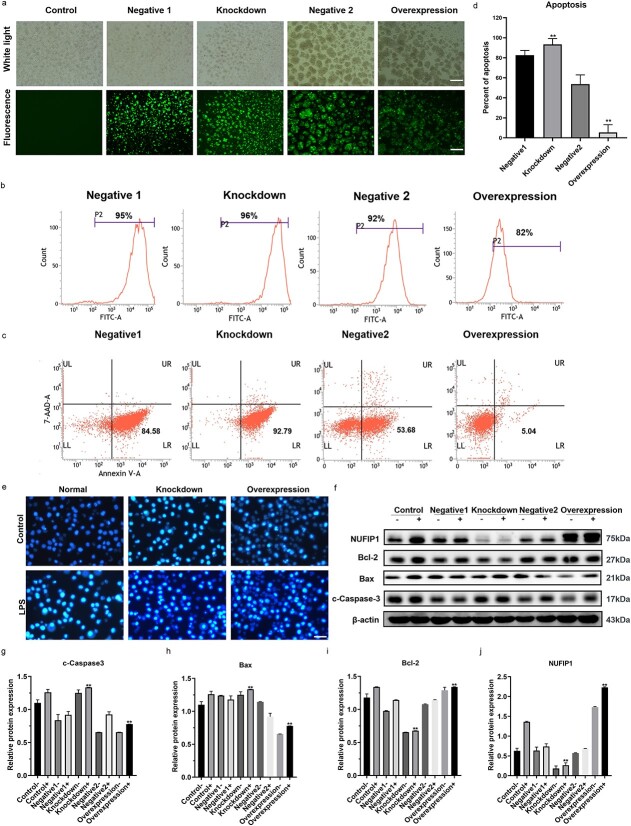
Protective effect of ribophagy on apoptosis in *Jurkat* cells under LPS stimulation. (**a**) The transfection of lentivirus was observed under a microscope (scale bar: 100 μm). (**b**) Flow cytometry revealed that the transfection efficiency of the NUFIP1-knockdown group and NUFIP1-overexpression group exceeded 80%. (**c, d**) Flow cytometric analysis showed that compared to the negative group, the NUFIP1-knockdown group showed an appreciable upregulation of apoptosis [Annexin V (+) cells %], while the overexpression group showed a marked anti-apoptotic response. (**e**) Hoechst 33258 was used to stain the normal group, knockdown group and overexpression group, and cell apoptosis was observed in each group under PBS and LPS stimulation with a fluorescence microscope (scale bar: 50 μm). (**f**–**j**) Western blot analysis showed that NUFIP1 expression was significantly reduced in the knockdown group and significantly enhanced in the overexpression group. Compared with that in the negative control group, the expression of c-Caspase-3 and Bax in the NUFIP1 overexpression group was significantly reduced, while Bcl-2 expression was significantly enhanced. In contrast, after knocking down the NUFIP1 gene, the expression levels of Caspase-3 and Bax in *Jurkat* cells were noticeably increased, while Bcl-2 expression was significantly reduced. One-way ANOVA was applied to test the statistical significance. Data are expressed as means ± SEM; ^*^^*^p < 0.01. *FITC* fluorescein isothiocyanate, *7-AAD* 7-aminoactinomycin D, *UL* upper left, *UR* upper right, *LL* lower left, *LR* lower right, *NUFIP1* nuclear fragile X mental retardation-interacting protein 1, *LPS* lipopolysaccharide, *PBS* phosphate buffer solution, *ANOVA* analysis of variance, *SEM* standard error of mean

After exploring the optimal time and LPS stimulation concentration for ribophagy by WB, we subsequently verified our results by observing NUFIP1 expression and colocalization through LSCM and autophagosome formation through TEM. The LSCM analysis showed that NUFIP1 was distributed in both the cytoplasm and nucleus and was predominantly located in the nucleus under normal conditions (Figure 2a–f). However, after treatment with 500 ng/ml LPS for 24 h, NUFIP1 expression was remarkably augmented in splenic CD4^+^ T lymphocytes and *Jurkat* cells. The translocation of NUFIP1 from the nucleus to the cytoplasm and the colocalization of NUFIP1 with LC-3B or LAMP-2 were obviously strengthened. When the LPS stimulation time was increased to 72 h, the above changes gradually decreased, becoming close to the levels observed before LPS stimulation (Figure 2a–d). As shown in Figure 2g**,** TEM imaging revealed that the number of autophagosomes that contained numerous ribosomes to be degraded was significantly increased, and the ER of splenic CD4^+^ T lymphocytes was appreciably swollen in the LPS-treated group. Similar to the *in vitro* experiments, LSCM examination of the CLP 24 h groups indicated that the expression of NUFIP1 and its colocalization with LC-3B or LAMP-2 were obviously strengthened, and TEM imaging showed that the formation of autophagosomes that contained numerous ribosomes to be degraded was also significantly enhanced (Figure 2e, f and h).

### Protective effect of ribophagy on T-lymphocyte apoptosis

To explore the effects of NUFIP1-mediated ribophagy on the apoptosis of T lymphocytes, *Jurkat* cells were transfected with lentiviral-based siRNA carrying a construct for NUFIP1 gene KD or overexpression. As shown in Figure 3a, b, fluorescence microscopy identified strong green fluorescence, and the transfection rates of *Jurkat* cells in the experimental groups measured by flow cytometry all exceeded 80%.

In this study, an annexin-V-PE/7-AAD quantification assay and Hoechst 33258 staining were utilized to assess the effects of ribophagy on apoptosis in various groups of stably transfected *Jurkat* cells. As shown in Figure 3c and d, the percentage of apoptotic cells in the NUFIP1-KD group was substantially increased, whereas it was significantly decreased in the NUFIP1-overexpression group compared with the negative groups (lentivirus empty vector groups), and the differences were statistically significant (*p* < 0.01). Furthermore, Hoechst 33258 staining showed that there were more condensed or fragmented apoptotic nuclei with high fluorescence intensity in the NUFIP1 KD group (Figure 3e). Moreover, it was revealed that the expression of NUFIP1 and the anti-apoptotic protein Bcl-2 was substantially reduced in the KD group, while c-Caspase-3 and Bax were significantly upregulated. In contrast, when the NUFIP1 gene was overexpressed, the expression of the above proteins showed the opposite trends. Namely, the expression levels of c-Caspase-3 and Bax were evidently decreased, while those of NUFIP1 and Bcl-2 were significantly increased compared with those in the negative control groups (Figure 3f–j).

**Figure 4:**
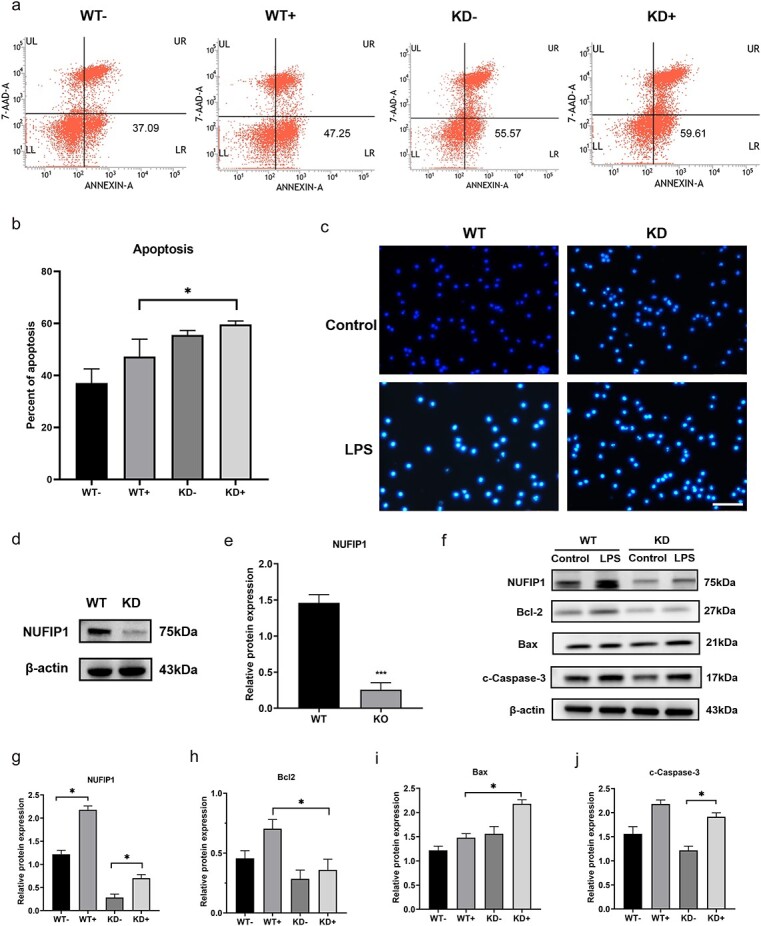
The impact of ribophagy on apoptosis in NUFIP1 gene-deficient mice after LPS stimulation. (**a, b**) Flow cytometric analysis showed that compared with that in WT mice, the apoptotic rate in NUFIP1 gene-deficient mice was significantly elevated. (**c**) Hoechst 33258 was used to stain CD4^+^ T lymphocytes in WT and NUFIP1 gene-deficient groups, and cell apoptosis was observed in each group under PBS and LPS stimulation with a fluorescence microscope (scale bar: 50 μm). (**d, e**) Western blot analysis showed that compared with that in WT mice, the NUFIP1 protein expression in the gene-deficient mice was meaningfully reduced. (**f**–**j**) Western blot analysis revealed that the expression levels of the proapoptotic proteins Caspase-3 and Bax were remarkably increased in the NUFIP1 gene-deficient group compared with the WT group, while the expression of Bcl-2 was significantly diminished (−: without LPS stimulation; +: with LPS stimulation). One-way ANOVA test (**b**, **g**, **h**, **i**, **j**); unpaired two-sided Student’s t test (e). Data are expressed as means ± SEM; ^*^p < 0.05. *7-AAD* 7-aminoactinomycin D, *UL* upper left, *UR* upper right, *LL* lower left, *LR* lower right, *WT* wild type, *KD* knockdown, *LPS* lipopolysaccharide, *NUFIP1* nuclear fragile X mental retardation-interacting protein 1, *PBS* phosphate buffer solution, *ANOVA* analysis of variance, *SEM* standard error of mean

To explore the protective effect of ribophagy on T lymphocytes, we constructed NUFIP1 gene-deficient mice to validate the abovementioned results. The results of flow cytometry and Hoechst 33258 apoptosis staining assays were similar to those found in the *in vitro* experiments: compared with that in WT mice, the percentage of apoptotic cells in NUFIP1 gene-deficient mice was significantly elevated (Figure 4a–c). Consistently, the expression levels of c-Caspase-3 and Bax were significantly increased in the NUFIP1-deficient group, while those of NUFIP1 and Bcl-2 were obviously reduced in this group compared with the WT group, and the differences were statistically significant (Figure 4d–j, *p* < 0.05).

### Impacts of ribophagy on the survival and systemic immune status of septic mice

**Figure 5:**
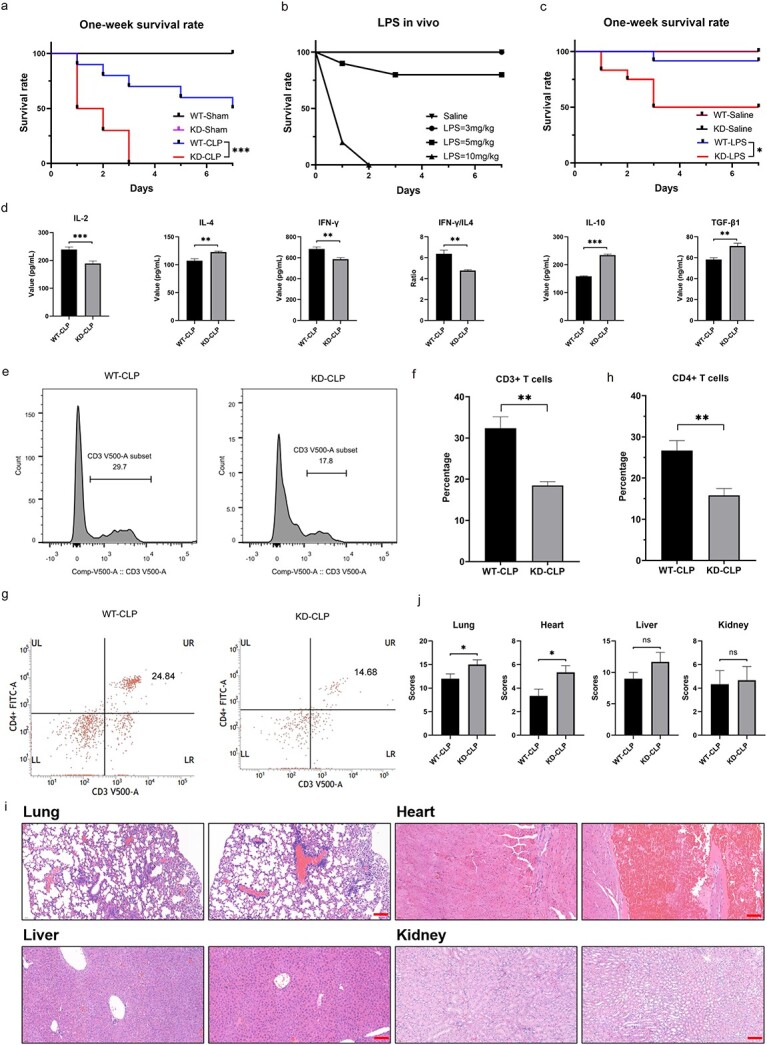
Effect of ribophagy on the survival rate and systemic immune status of NUFIP1 gene-deficient mice in sepsis. (**a**) The survival curves showed that the mortality rate of NUFIP1 gene-deficient mice was notably higher than that of WT mice 1 week after CLP (*n* = 10). (**b**) Following the injection of 3, 5 and 10 mg/kg LPS into 10 mice each, the survival curves showed that no mice died in the 3 mg/kg LPS group. Two mice in the 5 mg/kg LPS group died after intraperitoneal injection at 24 and 72 h. In the 10 mg/kg LPS group, 8 mice died after 24 h and all mice died after 48 h (*n* = 10). (**c**) The survival curves showed that the mortality rate of NUFIP1 gene-deficient mice was markedly higher than that of WT mice 1 week after intraperitoneal LPS injection (*n* = 10). (**d**) Quantitative bar charts showing the levels of multiple cytokines, including interleukin (IL)-2, IL-4, IFN-γ, ratio of IFN-γ to IL-4, IL-10 and TGF-β1, in mouse serum for the WT-CLP and KD-CLP groups. (**e**–**h**) Flow cytometric analysis showed that the total number of CD3 cells and CD3/CD4 double-positive cells in the KD-CLP group were significantly lower than those in WT-CLP mice. (**i, j**) Histological scores (right panel) and representative images of hematoxylin and eosin (HE) staining (left panel) elucidating the pathological alterations in multiple organs of mice that underwent sham or CLP surgery, including the lung, liver, kidney and heart (scale bar: 50 μm). Unpaired two-sided Student’s t test (d, f, h, j**)**. Data are expressed as means ± SEM; ^*^p < 0.05, ^*^^*^p < 0.01, ^*^^*^^*^p < 0.001. *WT* wild type, *KD* knockdown, *LPS* lipopolysaccharide, *CLP* cecal ligation and puncture, *IL* interleukin, *IFN-γ* interferon-γ, *TGF-β1* transforming growth factor-β1, *UL* upper left, *UR* upper right, *LL* lower left, *LR* lower right, *NUFIP1* nuclear fragile X mental retardation-interacting protein 1, *PBS* phosphate buffer solution, *HE* hematoxylin and eosin, *SEM* standard error of mean

To investigate the impact of ribophagy on the systemic pathophysiological changes in septic mice, we evaluated the 1-week survival rate of WT and NUFIP1 gene-deficient mice after CLP. The 1-week Kaplan–Meier curves showed that the mortality rate of NUFIP1 gene-deficient mice was remarkably higher than that of WT mice (Figure 5a, *p* < 0.001). Additionally, we constructed an endotoxin model by injecting LPS intraperitoneally into WT and NUFIP1-deficient mice to make the results more complete and clinically translatable. Consistently, the 7-day survival rate of NUFIP1-deficient mice was significantly lower than that of WT mice, and the difference was statistically significant (Figure 5b, c, *p* < 0.05). Moreover, our analyses showed that NUFIP1 gene-deficient mice were significantly associated with elevated immunosuppression of T lymphocytes upon septic challenge, including the decreased level of IL-2, IFN-γ, ratio of IFN-γ to IL-4 and increased level of IL-4, IL-10 and TGF-β1, as shown in Figure 5d. Moreover, flow cytometric analysis showed that the total number of CD3 cells and CD3/CD4 double-positive cells in NUFIP1 gene-deficient mice were significantly lower than those in WT mice (Figure 5e–h). Furthermore, at 24 h after sepsis onset, the deficiency of NUFIP1 was correlated with injuries to multiple organs, which were determined by histological examination using a standardized scoring system (Figure 5i, j).

**Figure 6:**
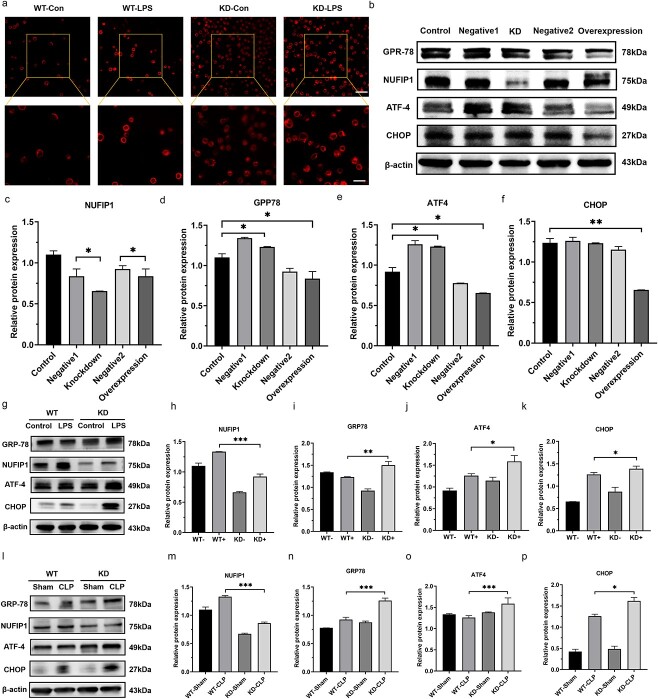
Ribophagy protected T lymphocytes from an excessive ERS response in sepsis. (**a**) LSCM was used to observe the changes in the ER structure after knocking down the NUFIP1 gene. Fragmentation and swelling of ER in splenic CD4^+^ T lymphocytes were observed after LPS stimulation compared with control treatment. These alterations were more pronounced in NUFIP1-deficient mice [scale bars, 25 μm (top row) and 10 μm (bottom row)]. (**b**–**f**) The expression of ERS apoptosis-associated proteins was identified by WB in *Jurkat* cells. The results showed that compared with the negative control group, the NUFIP1-KD group showed markedly increased expression levels of GRP78, CHOP and ATF4. In contrast, when the NUFIP1 gene was overexpressed, the expression levels of GRP78, CHOP and ATF4 in *Jurkat* cells were significantly reduced. (**g**–**k**) The expression of ERS apoptotic-associated proteins was identified by WB in CD4^+^ T lymphocytes. (**l**–**p**) CLP models were constructed with WT mice and NUFIP1-deficient mice. Compared with the sham procedure, CLP alone induced the expression of NUFIP1, and the protein expression levels of GRP78, ATF4 and CHOP were obviously increased. The expression of the NUFIP1 protein was significantly lower in the NUFIP1-deficient group than in the WT group, and the expression levels of GRP78, ATF4 and CHOP increased more significantly. One-way ANOVA was applied to test the statistical significance. Data are expressed as means ± standard error of mean; ^*^p < 0.05, ^*^^*^p < 0.01, ^*^^*^^*^p < 0.001. *WT* wild type, *KD* knockdown, *LPS* lipopolysaccharide, *CLP* cecal ligation and puncture, *NUFIP1* nuclear fragile X mental retardation-interacting protein 1, *LSCM* laser scanning confocal microscopy, *ER* endoplasmic reticulum, *RES* endoplasmic reticulum stress, *GRP78* glucose-regulated protein 78, *ATF4* activating transcription factor 4, *CHOP* C/EBP homologous protein, *UL* upper left, *UR* upper right, *LL* lower left, *LR* lower right, *ANOVA* analysis of variance

**Figure 7:**
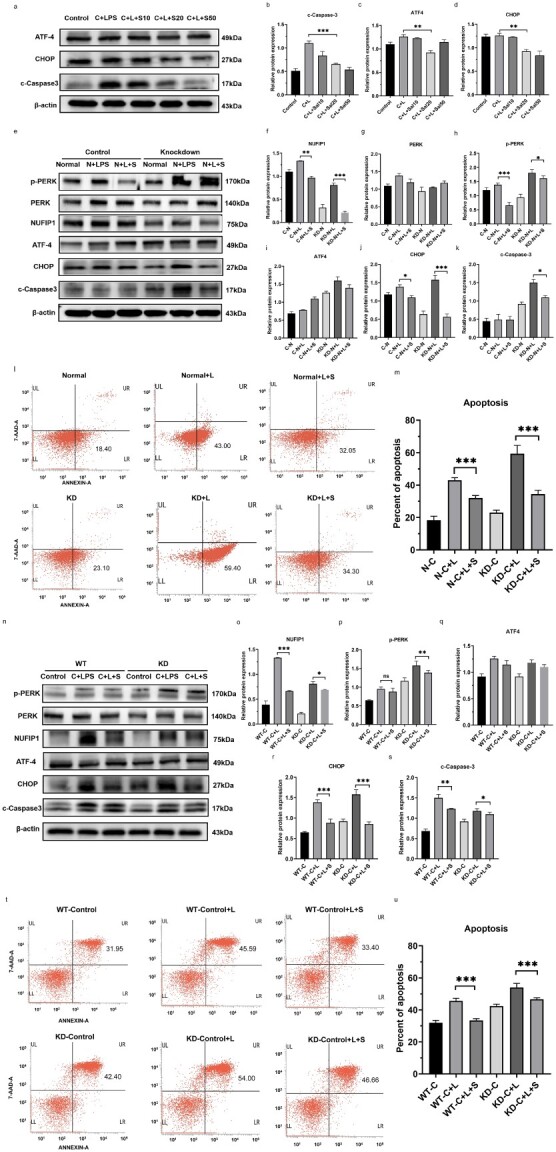
Ribophagy downregulated the PERK–ATF4–CHOP signaling pathway to alleviate ERS-related cell apoptosis in T lymphocytes. (**a**–**d**) *Jurkat* cells were pretreated with various concentrations of the upstream inhibitor salubrinal (Sal, 10, 20, 50 mM) for 2 h and then subjected to treatment with 500 ng/ml LPS for 24 h. Pretreatment with Sal, particularly at 20 mM, notably diminished LPS-induced ATF4 and CHOP expression and apoptosis. (**e-k**) *Jurkat* cells were pretreated with 20 mM Sal for 2 h and then treated with 500 ng/ml LPS for 24 h. Pretreatment with Sal markedly weakened LPS-induced ATF4 and CHOP expression and apoptosis. (**l**, **m**) Normal and NUFIP1-KD *Jurkat* cells were pretreated with 20 mM Sal for 2 h and then exposed to 500 ng/ml LPS for 24 h. Flow cytometric analysis showed that pretreatment with Sal obviously inhibited apoptosis compared with LPS alone. (**n**–**s**) The expression of PERK–ATF4–CHOP signaling pathway-related proteins in Splenic CD4^+^ T lymphocytes from WT and NUFIP1-deficient mice that were pretreated with 20 mM Sal for 2 h and then subjected to 500 ng/ml LPS for 24 h. (**t**, **u**) Flow cytometric analysis showed the apoptosis of Splenic CD4^+^ T lymphocytes in WT and NUFIP1-deficient mice with LPS stimulation or LPS plus Sal. One-way ANOVA was applied to test the statistical significance. Data are expressed as means ± SEM; ^*^p < 0.05, ^*^^*^p < 0.01, ^*^^*^^*^p < 0.001. *C* control group, *L* lipopolysaccharide, *S* salubrinal, *N* normal, *WT* wild type, *KD* knockdown, *CLP* cecal ligation and puncture, *NUFIP1* nuclear fragile X mental retardation-interacting protein 1, *RES* endoplasmic reticulum stress, *PERK* protein kinase RNA-like ER kinase, *GRP78* glucose-regulated protein 78, *ATF4* activating transcription factor 4, *CHOP* C/EBP homologous protein, *7-AAD* 7-aminoactinomycin D, *UL* upper left, *UR* upper right, *LL* lower left, *LR* lower right, *ANOVA* analysis of variance, *SEM* standard error of mean

### Ribophagy protects T lymphocytes from an excessive endoplasmic reticulum stress response during sepsis

Given the close functional and structural relationship between the ribosome and ER, we hypothesized that ER stress (ERS)-related apoptotic signaling might be the main pathway through which ribophagy alleviates T-lymphocyte apoptosis. We first applied LSCM to observe the alterations in the ER structure after knocking down the NUFIP1 gene. As shown in Figure 6a, fragmentation and swelling changes in the ER of splenic CD4^+^ T lymphocytes were noticed after LPS stimulation compared with control treatment. These alterations and fragmentation were more pronounced in NUFIP1-deficient mice.

To further test our hypothesis, we treated stably transfected and nontransfected *Jurkat* cells with 500 ng/ml LPS for 24 h and then measured the expression of the unfolded protein response (UPR)-related proteins GRP78, ATF4 and CHOP by western blot analysis. As shown in Figure 6b–f, the expression levels of GRP78, CHOP and ATF-4 were significantly enhanced in the NUFIP1-KD group (*p* < 0.05). In contrast, when the NUFIP1 gene was overexpressed, the expression levels of these UPR-related proteins were consistently reduced. After LPS-stimulation for 24 h, the expression levels of the UPR apoptosis-related proteins GRP78, CHOP and ATF4 were significantly upregulated in NUFIP1-deficient mice compared with WT mice, and the differences were statistically significant (Figure 6g–k, *p* < 0.05). *In vivo*, the expression levels of GRP78, CHOP and ATF4 were also significantly augmented in WT mice that underwent CLP surgery compared with mice in the sham group, and the differences were magnified in NUFIP-1-deficient mice (Figure 6l–p).

### Ribophagy ameliorates UPR-related cell apoptosis in a PERK–ATF4–CHOP-dependent manner

To further understand the possible signaling pathway related to the protective impact of ribophagy on UPR-related apoptosis, *Jurkat* cells were pretreated with different concentrations of the eIF2a inhibitor Sal (10–50 mM) for 2 h and then exposed to 500 ng/ml LPS for 24 h. As shown in Figure 7a–d, pretreatment with Sal significantly weakened the expression of ATF4, CHOP and c-Caspase 3, especially at 20 mM. WB results showed that knocking down NUFIP1 aggravated the activation of caspase 3, which was in line with the upregulation of the expression of p-PERK, ATF4 and CHOP. However, these changes were significantly attenuated by pretreatment with Sal to block the PERK–ATF4–CHOP signaling pathway, and these effects were presented in both the NUFIP1 KD and control groups (Figure 7e–k). As shown in Figure 7l and m**,** both the control and NUFIP1 KD groups exhibited obviously increased apoptosis rates after LPS stimulation, which could be reduced by pretreatment with Sal. We subsequently stimulated splenic CD4^+^ T lymphocytes from WT and NUFIP1 gene-deficient mice with LPS to confirm the above western blot analysis and flow cytometry results. The results showed that blocking the PERK–ATF4–CHOP signaling pathway with Sal effectively alleviated the apoptosis of splenic CD4^+^ T lymphocytes (Figure 7n–u).

**Figure 8:**
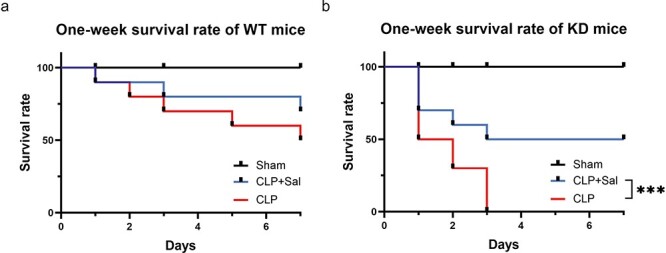
Improvement in the 1-week survival rate of NUFIP1 gene-deficient mice subjected to CLP with salubrinal pretreatment. (**a**) The 1-week survival curves showed that the mortality rate of WT mice pretreated with 2 mg/kg Sal was lower than that of WT mice 1 week after CLP (*n* = 10). (**b**) The 1-week survival curves of NUFIP1 gene-deficient mice (*n* = 10); ^*^p < 0.05, ^*^^*^p < 0.01, ^*^^*^^*^p < 0.001. *WT* wild type, *KD* knockdown, *CLP* cecal ligation and puncture, *Sal* Salubrinal, *NUFIP1* nuclear fragile X mental retardation-interacting protein 1

Finally, we evaluated the influence of pretreatment with Sal on the 1-week survival rate of CLP mice. As shown in Figure 8a, pretreatment with Sal (2 mg/kg) 1 h prior to CLP improved the 7-day mortality in WT mice, although the difference was not statistically significant. In contrast, Sal pretreatment significantly improved the 1-week postoperative survival rate of NUFIP-deficient CLP mice (Figure 8b, *p* < 0.001), which suggested that NUFIP1-mediated ribophagy might inhibit overactivation of the PERK–ATF4–CHOP signaling pathway to ameliorate the apoptosis of T lymphocytes following septic challenge.

## Discussion

Despite considerable efforts to improve the prevention and treatment of sepsis, millions of individuals worldwide develop septic complications each year and even progress to fatal outcomes [24]. It is well documented that immunosuppression is the primary cause of death in septic patients, and cellular dysfunction appears to be the pathological basis and key link of sepsis-induced immune dysregulation [25]. As a pivotal component of the adaptive immune response, T lymphocytes mediate the core of ‘cellular immunity’ [26,27]. Therefore, effective maintenance of the immune function of T lymphocytes is critical in the host [28,29]. A body of studies has indicated that inordinate apoptosis or immune nonresponsiveness in T lymphocytes ordinarily leads to an immunosuppressive state, thus increasing host susceptibility to septic challenge and aggravating disease conditions [30–33]. Schmidt *et al*. found that massive apoptosis of T lymphocytes resulted in irreversible damage and loss of cell function, which ultimately led to the deterioration of septic patients [34]. Further studies by Rittirsch *et al*. and Tang *et al*. revealed that regulating or reversing the apoptosis and polarization of T lymphocytes could efficaciously ameliorate the sepsis-induced immunosuppressive status [35,36]. Therefore, effective maintenance of the number and function of T lymphocytes is a key approach to diminishing the immune dysfunction in sepsis and is worthy of further research.

**Figure 9:**
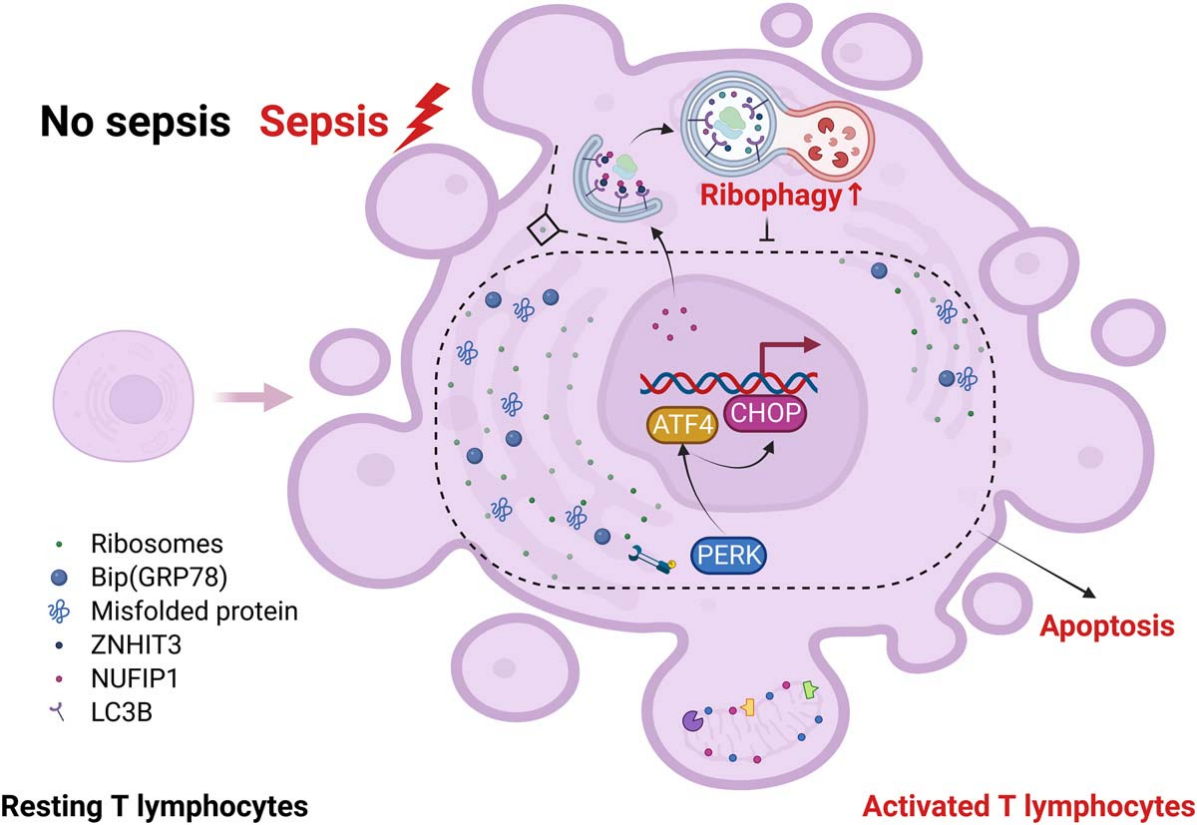
Schematic diagram showing the mechanism by which NUFIP1-mediated ribophagy protects T lymphocytes against apoptosis following septic challenge. In T lymphocytes, NUFIP1-mediated ribophagy was remarkably upregulated and functioned as a protective mechanism against apoptosis via the PERK–ATF4–CHOP pathway in the context of sepsis. *NUFIP1* nuclear fragile X mental retardation-interacting protein 1, *PERK* protein kinase RNA-like ER kinase, *GRP78* glucose-regulated protein 78, *ATF4* activating transcription factor 4, *CHOP* C/EBP homologous protein, *ZHHIT3* zinc finger HIT domain containing protein 3, *LC-3B* light chain 3B

Of note, septic patients can manifest various metabolic abnormalities, including increased peripheral glucose intake and demand for calories as well as protein. In other words, under septic conditions, cells are in a state of nutritional deficiency characterized by a lack of calories, proteins and amino acids, which is similar to Wyant’s cell model of starvation [18]. Since starvation can induce the occurrence of ribophagy, we had sufficient reasons to believe that sepsis is likely to trigger the upregulation of ribophagy. In the current study, we first investigated the activity of ribophagy in T lymphocytes in the setting of sepsis. The results indicated that CLP and LPS-stimulated sepsis could both effectively activate ribophagy, indicating that sepsis is an important stimulator of ribophagy in addition to nutritional deficiency and mammalian target of rapamycin inhibitor treatment.

Ribosomes are mainly responsible for RNA translation and protein folding. Most ribosomes are attached to the rough ER in cells; they actively synthesize antibodies, proenzymes and proteins and transport them outside the cell [37]. A small number of free ribosomes are present in the cell matrix, binding to the cytoskeleton and providing proteins for cell growth [38]. Although ribosomes may have translation folding errors or be exposed to various stresses from inside and outside the cell [39,40], as precision-assembled organelles, they have evolved a variety of RQCS, including ribophagy, to counter and alleviate ribosomal dysfunction. Since ribophagy was first proposed to occur in *S. cerevisiae* by Kraft *et al*. in 2008 [17], ribophagy-related research has progressed slowly and its correlation with human diseases is rarely reported. More recently, Wyant *et al*. identified that NUFIP1 combined with Zinc finger HIT domain containing protein 3 (ZHHIT3) served as a specific receptor for ribophagy, which provided an unambiguous interventional target for subsequent related research [18]. Normally, NUFIP1 exists in the nucleus and acts as an important synthesizer of snoRNP [41]. Although cells could express NUFIP1 under physiological conditions, its expression in sepsis was appreciably upregulated. Moreover, LSCM showed that NUFIP1 was ordinarily concentrated in the nucleus, while the shuttling of NUFIP1 from the nucleus to the cytoplasm could be clearly observed in the context of sepsis. This was consistent with a previous study showing that NUFIP1 needed to travel to the cytoplasm and to act as a receptor in ribophagy. In addition, TEM results showed that splenic CD4^+^ T lymphocytes in sepsis exhibited notable formation of autophagosomes containing copious ribosomes to be degraded and swelling of the ER, which further confirmed the conspicuous phenomenon of ribophagy in T lymphocytes induced by septic challenge.

Previous reports have documented that mitophagy and ER-phagy are remarkably upregulated in sepsis and can sustain the normal function of the corresponding organelles, thereby significantly improving the poor prognosis of sepsis [21,22]. Can ribophagy play a protective role analogous to other organelles in sepsis? Based on this idea, we tried to explore the impact of ribophagy on the apoptosis of T lymphocytes in sepsis. We constructed NUFIP1-KD and NUFIP1-overexpression models with *Jurkat* cells and the results indicated that the apoptosis of LPS-stimulated cells was markedly increased after knocking down NUFIP1, while the overexpression of NUFIP1 had a meaningful mitigating impact on T-lymphocyte apoptosis. Identical results were obtained *in vivo* for NUFIP1-deficient mice, and the apoptotic rate of NUFIP1-deficient mice was obviously elevated compared with that of WT mice after CLP. Moreover, NUFIP1-deficiency attenuated the 1-week survival rate and systemic immune status of CLP mice. Hence, further elucidation of the significance and associated underlying mechanism of NUFIP1-mediated ribophagy involved in T-lymphocyte apoptosis is expected to uncover a novel interventional target for treatment.

ERS is a devastating state of ER homeostasis that can be induced by various factors, such as ischemia, hypoxia, adenosine triphosphate depletion, Ca^2+^ balance disorder, abnormal increase in new synthetic proteins or disordered protein processing, modification and transport [42]. Mild ERS can reinstate normal protein processing and promote cell survival by activating the UPR, however, if ERS exceeds the conservation capacity of the UPR, ER dysfunction or even death of the stressed cell may occur [43]. It was reported that in a mouse model of sepsis, excessive ERS aggravated lymphocyte apoptosis and was one of the critical mechanisms in the deterioration of sepsis [44]. Our previous study suggested that the activation of sestrin2 (SESN2) in dendritic cells after high mobility group box-1 protein stimulation protected against apoptosis by regulating the extent of the ERS response and that inhibition of ERS noticeably ameliorated immune dysfunction and improved the 7-day survival rate of CLP mice [45]. Herein, we found that the apoptosis of T lymphocytes in sepsis was closely related to UPR-related proteins, including GRP78, ATF4 and CHOP. Both *in vitro* data and *in vivo* results consistently indicated that the expression levels of GRP78, CHOP and ATF4 were significantly enhanced after knocking down or silencing NUFIP1. In contrast, when NUFIP1 was overexpressed, the expression levels of these UPR-related proteins were obviously reduced. Considering the tight structural and functional interrelation between ribosomes and ER, all these responses seemed justifiable.

The PERK–ATF4 signaling pathway is one of the indispensable ERS signaling pathways in addition to the IRE1–XBP1 and ATF6 pathways, all of which can lead to the transcription of CHOP, a pivotal mediator in the development of sepsis [46]. Emerging evidence suggests that the PERK–ATF4–CHOP pathway is related to the activation of cytoprotective autophagy upon ERS in different cellular models [47–49]. Our previous studies demonstrated that the PERK–ATF4–CHOP signaling pathway was critical for SESN2 alleviating the apoptosis, pyroptosis and ferroptosis of dendritic cells in sepsis [45,46,50]. In view of signaling in immune cells and the close interplay among multiple organelles, we concluded that certain signaling pathways might be involved in the regulation of ribophagy in T lymphocytes secondary to septic exposure. Thus, we investigated whether the PERK–ATF4–CHOP signaling pathway contributes to NUFIP1-mediated ribophagy with a protective effect on the apoptosis of T lymphocytes in sepsis. The experimental results from this study confirmed our hypothesis, as evidenced by the observation that blocking the PERK–ATF4–CHOP signaling pathway with Sal could effectively alleviate the apoptosis of T lymphocytes. Strikingly, pretreatment with Sal significantly improved 1-week postoperative survival in NUFIP1-deficient CLP mice, suggesting that NUFIP1-mediated ribophagy might ameliorate UPR-related cell apoptosis, at least in part, in a PERK–ATF4–CHOP-dependent manner (Figure 9).

Nevertheless, some limitations remained in our study. First, since a lentivirus itself has a certain effect on cell apoptosis, which confounded our results to a certain extent, we tried to reduce this effect by strict grouping corresponding to this principle. Second, we substantiated the proposed role for NUFIP1-mediated ribophagy in protecting T lymphocytes against apoptosis via the PERK–ATF4–CHOP pathway, but the definite mechanism underlying this signaling is still obscure. Third, due to homozygous death in NUFIP1 gene-defective mice, we selected heterozygous mice for some of the experiments. Although we have demonstrated the feasibility of heterozygous mice at the protein level, subsequent verification in conditional knockout mice is needed in the future. Finally, clinical or preclinical studies investigating the implications of ribophagy in treating human diseases should be performed in the foreseeable future.

## Conclusions

Overall, we propose that NUFIP1-mediated ribophagy remarkably functions as a protective mechanism against T-lymphocyte apoptosis mediated through the PERK–ATF4–CHOP pathway in the context of sepsis. The present study first confirms that ribophagy may act as a quality control method to maintain the functional homeostasis of T lymphocytes and even improve the 1-week survival rate in CLP-induced sepsis. Recently, studies on organelle interactions have attracted increasing attention from researchers. In our data, we noticed the close association between ribosomes and the ERS-related apoptosis pathway, which might provide new ideas for follow-up studies on the relationship between ribosomes and the ER. Finally, yet importantly, as NUFIP1 is the unique receptor currently available for ribophagy, targeting NUFIP1-mediated ribophagy might be of importance in reversing the sepsis-induced immunosuppressive state in critical illnesses.

## Abbreviations

7-AAD: 7-Aminoactinomycin D; ANOVA: Analysis of variance; ATF4: Activating transcription factor 4; CHOP: C/EBP homologous protein; CLP: Cecal ligation and puncture; Con A: Concanavalin A; DAPI: 4′,6-Diamino-nuclei 2-phenylindole; EDTA: Ethylene diamine tetraacetic acid; ER: Endoplasmic reticulum; ERS: Endoplasmic reticulum stress; FITC: Fluorescein isothiocyanate; GRP78: Glucose-regulated protein 78; HE: Hematoxylin–eosin; IFN-γ: Interferon-γ; IL: Interleukin; KD: Knockdown; LAMP2: Lysosomal associated membrane protein 2; LC-3B: Light chain 3B; LPS: Lipopolysaccharide; LSCM: Laser scanning confocal microscopy; NUFIP1: Nuclear fragile X mental retardation-interacting protein 1; PBS: Phosphate buffered saline; PBMCs: Peripheral blood mononuclear cells; PE: Phycoerythrin; PERK: Protein kinase RNA-like ER kinase; RPL7: Ribosomal protein L7; RQCS: ribosome quality control systems; Sal: Salubrinal; SDS: Sodium dodecyl sulfate; SESN2: Sestrin2; siRNA: Small interfering RNA; TEM: Transmission electron microscopy; TGF-β1: Transforming growth factor-β1; TUNEL: Terminal-deoxynucleoitidyl transferase mediated nick end labeling; UPR: Unfolded protein response; WB: Western blotting; WT: Wild-type; ZHHIT3: Zinc finger HIT domain containing protein 3.

## Supplementary Material

Supplementary_data_1_tkac055

Supplementary_data_2_tkac055

Supplementary_data_3_tkac055

Figure_S3_tkac055

## Data Availability

The data and material is available from the corresponding authors on reasonable request.
