# Supplementary material for: Nuclear fragile X mental retardation-interacting protein 1-mediated ribophagy protects T lymphocytes against apoptosis in sepsis
Source: Burns Trauma. 2023 Feb 28;11:tkac055. doi: 10.1093/burnst/tkac055 (PMC9976742; doi:10.1093/burnst/tkac055)
Supplement: Supplementary_data_1_tkac055 [file supplementary_data_1_tkac055.docx]

### Supplementary data 1. Off target analysis

In the present study, we searched the off-target sites that exactly matches 12–13 bases at the 3′ end and the NGG (N can be A, G, C, or T) with free software, COSMID (https://crispr.bme.gatech.edu/) against whole mouse genomic sequence (mm10). The 10 potential off-target sites with the highest scores (highest off-target potential) were screened and PCR amplification primers were derived to target these off-target sites. The ~900bp genomic fragments containing the off-target in the center were PCR amplified and sequenced.

| gRNA1 | TAACCAAACACAAAGACCCC AGG |
| --- | --- |

The following five off-target detection sites were designed for gRNA1

1#

| **Result** | **Query type** | **Mismatch** | **Hit ends in RG** | **chr position** | **Strand** | **Cut site** | **Score** |
| --- | --- | --- | --- | --- | --- | --- | --- |
| AAACAAAACAAAAAGACCCCTGG -- hit TAACCAAACACAAAGACCCCNGG -- query | No indel | 3 | Yes | [Chr3:54735968-54735990](http://genome.ucsc.edu/cgi-bin/hgTracks?org=Human&db=mm10&position=Chr3:54735968-54735990&hgt.suggestTrack=knownGene) | - | 54735974 | 1.01 |

PCR Primers

| F1（831bp） | CCTTGGCCTCAGATTCCCTG |
| --- | --- |
| R1 | AATGCCAACAGGCAGATGGA |

Sequencing Primers

| ID-F1 | CCTTGGCCTCAGATTCCCTG |
| --- | --- |
| ID-R1 | AATGCCAACAGGCAGATGGA |

2#

| **Result** | **Query type** | **Mismatch** | **Hit ends in RG** | **chr position** | **Strand** | **Cut site** | **Score** |
| --- | --- | --- | --- | --- | --- | --- | --- |
| AAACGAAACACAAAGACCCCTTG -- hit TAACCAAACACAAAGACCCCNGG -- query | No indel | 3 | No | [Chr19:38751857-38751879](http://genome.ucsc.edu/cgi-bin/hgTracks?org=Human&db=mm10&position=Chr19:38751857-38751879&hgt.suggestTrack=knownGene) | - | 38751863 | 20.31 |

PCR Primers

| F2（684bp） | ATCTGGTGCTGGGAGGATCT |
| --- | --- |
| R2 | AGGGCATTGGCTCTCCTAGA |

Sequencing Primers

| ID-F2 | ATCTGGTGCTGGGAGGATCT |
| --- | --- |
| ID-R2 | AGGGCATTGGCTCTCCTAGA |

3#

| **Result** | **Query type** | **Mismatch** | **Hit ends in RG** | **chr position** | **Strand** | **Cut site** | **Score** |
| --- | --- | --- | --- | --- | --- | --- | --- |
| AAACCAAACCCAAAGTCCCCAGG -- hit TAACCAAACACAAAGACCCCNGG -- query | No indel | 3 | Yes | [Chr5:120408778-120408800](http://genome.ucsc.edu/cgi-bin/hgTracks?org=Human&db=mm10&position=Chr5:120408778-120408800&hgt.suggestTrack=knownGene) | - | 120408784 | 2.92 |

PCR Primers

| F3（837bp） | CTTGAACTGCCTATGCCCCA |
| --- | --- |
| R3 | CGTCCTCACCAATGTCACCA |

Sequencing Primers

| ID-F3 | CTTGAACTGCCTATGCCCCA |
| --- | --- |
| ID-R3 | CGTCCTCACCAATGTCACCA |

4#

| **Result** | **Query type** | **Mismatch** | **Hit ends in RG** | **chr position** | **Strand** | **Cut site** | **Score** |
| --- | --- | --- | --- | --- | --- | --- | --- |
| CAACCCAACACAAAGACCCATGG -- hit TAACCAAACACAAAGACCCCNGG -- query | No indel | 3 | Yes | [Chr6:50235122-50235144](http://genome.ucsc.edu/cgi-bin/hgTracks?org=Human&db=mm10&position=Chr6:50235122-50235144&hgt.suggestTrack=knownGene) | + | 50235138 | 6.33 |

PCR Primers

| F4（780bp） | CAGCCAGGCCAGAAAAAGTG |
| --- | --- |
| R4 | TCCTCACACGCTAGGCTACT |

Sequencing Primers

| ID-F4 | CAGCCAGGCCAGAAAAAGTG |
| --- | --- |
| ID-R4 | TCCTCACACGCTAGGCTACT |

5#

| **Result** | **Query type** | **Mismatch** | **Hit ends in RG** | **chr position** | **Strand** | **Cut site** | **Score** |
| --- | --- | --- | --- | --- | --- | --- | --- |
| GAACGAAACACACAGACCCCAGG -- hit TAACCAAACACAAAGACCCCNGG -- query | No indel | 3 | Yes | [Chr7:118079022-118079044](http://genome.ucsc.edu/cgi-bin/hgTracks?org=Human&db=mm10&position=Chr7:118079022-118079044&hgt.suggestTrack=knownGene) | - | 118079028 | 1.41 |

PCR Primers

| F5（944bp） | TGCCACAACTCCCCATCATC |
| --- | --- |
| R5 | GATTAAGAGCGCTTGGCTGC |

Sequencing Primers

| ID-F5 | TGCCACAACTCCCCATCATC |
| --- | --- |
| ID-R5 | GATTAAGAGCGCTTGGCTGC |

| Grna2 | GGTGTTGCAACATCATGGCC GGG |
| --- | --- |

The following five off-target detection sites were designed for Grna2

1#

| **Result** | **Query type** | **Mismatch** | **Hit ends in RG** | **chr position** | **Strand** | **Cut site** | **Score** |
| --- | --- | --- | --- | --- | --- | --- | --- |
| AGTGTTGCAAGATCCTGGCCAGG -- hit GGTGTTGCAACATCATGGCCNGG -- query | No indel | 3 | Yes | [Chr19:10447707-10447729](http://genome.ucsc.edu/cgi-bin/hgTracks?org=Human&db=mm10&position=Chr19:10447707-10447729&hgt.suggestTrack=knownGene) | - | 10447713 | 2.72 |

PCR Primers

| F1（845bp） | GAGTTGCACAGAGGAGCCTT |
| --- | --- |
| R1 | TCGAGGTCAGTGTGTGCTTC |

Sequencing Primers

| ID-F1 | GAGTTGCACAGAGGAGCCTT |
| --- | --- |
| ID-R1 | TCGAGGTCAGTGTGTGCTTC |

2#

| **Result** | **Query type** | **Mismatch** | **Hit ends in RG** | **chr position** | **Strand** | **Cut site** | **Score** |
| --- | --- | --- | --- | --- | --- | --- | --- |
| GGT^TTGTAAGATCATGGCCTGG -- hit GGTGTTGCAACATCATGGCCNGG -- query | Del 17 | 2 | Yes | [Chr4:9574672-9574693](http://genome.ucsc.edu/cgi-bin/hgTracks?org=Human&db=mm10&position=Chr4:9574672-9574693&hgt.suggestTrack=knownGene) | + | 9574687 | 1.65 |

PCR Primers

| F2（915bp） | AGCTATGCAAAGCAGGAGCA |
| --- | --- |
| R2 | CTATGCAACAGGGGTGGGTT |

Sequencing Primers

| ID-F2 | AGCTATGCAAAGCAGGAGCA |
| --- | --- |
| ID-R2 | CTATGCAACAGGGGTGGGTT |

3#

| **Result** | **Query type** | **Mismatch** | **Hit ends in RG** | **chr position** | **Strand** | **Cut site** | **Score** |
| --- | --- | --- | --- | --- | --- | --- | --- |
| GTGGTTGCAGCATCATGGCCTGG -- hit GGTGTTGCAACATCATGGCCNGG -- query | No indel | 3 | Yes | [Chr14:14950846-14950868](http://genome.ucsc.edu/cgi-bin/hgTracks?org=Human&db=mm10&position=Chr14:14950846-14950868&hgt.suggestTrack=knownGene) | + | 14950862 | 0.78 |

PCR Primers

| F3（976bp） | CCTTGGCTCTCGTGGACTTT |
| --- | --- |
| R3 | CCCTGGTTTCTGGTAGGCAG |

Sequencing Primers

| ID-F3 | CCTTGGCTCTCGTGGACTTT |
| --- | --- |
| ID-R3 | CCCTGGTTTCTGGTAGGCAG |

4#

| **Result** | **Query type** | **Mismatch** | **Hit ends in RG** | **chr position** | **Strand** | **Cut site** | **Score** |
| --- | --- | --- | --- | --- | --- | --- | --- |
| GGTGTTTCAACATCATGGTCTGT -- hit GGTGTTGCAACATCATGGCCNGG -- query | No indel | 3 | No | [ChrX:82548204-82548226](http://genome.ucsc.edu/cgi-bin/hgTracks?org=Human&db=mm10&position=ChrX:82548204-82548226&hgt.suggestTrack=knownGene) | + | 82548220 | 25.23 |

PCR Primers

| F4（876bp） | ACATGCATGGGTTACCTCGT |
| --- | --- |
| R4 | AGTAGGTTCTGGCAATGGGC |

Sequencing Primers

| ID-F4 | ACATGCATGGGTTACCTCGT |
| --- | --- |
| ID-R4 | AGTAGGTTCTGGCAATGGGC |

5#

| **Result** | **Query type** | **Mismatch** | **Hit ends in RG** | **chr position** | **Strand** | **Cut site** | **Score** |
| --- | --- | --- | --- | --- | --- | --- | --- |
| GGTGTTGCAACACCTTTGCCCGG -- hit GGTGTTGCAACATCATGGCCNGG -- query | No indel | 3 | Yes | [Chr9:31898347-31898369](http://genome.ucsc.edu/cgi-bin/hgTracks?org=Human&db=mm10&position=Chr9:31898347-31898369&hgt.suggestTrack=knownGene) | + | 31898363 | 6 |

PCR Primers

| F5（872bp） | GGAGCTCTTGTTCCAGGCAT |
| --- | --- |
| R5 | CCAACCCTTGACACAGGCTA |

Sequencing Primers

| ID-F5 | GGAGCTCTTGTTCCAGGCAT |
| --- | --- |
| ID-R5 | CCAACCCTTGACACAGGCTA |

Abbreviations: *PCR* polymerase chain reaction, *RNA* ribose nucleic acid, *ID* identity.
