# Supplementary material for: Nuclear fragile X mental retardation-interacting protein 1-mediated ribophagy protects T lymphocytes against apoptosis in sepsis
Source: Burns Trauma. 2023 Feb 28;11:tkac055. doi: 10.1093/burnst/tkac055 (PMC9976742; doi:10.1093/burnst/tkac055)
Supplement: Supplementary_data_2_tkac055 [file supplementary_data_2_tkac055.docx]

**Supplemental data 2.** Criteria for the microscopic scoring of organ injuries.

| **Organ injuries** | **Scoring items** |
| --- | --- |
| **Lung injury** | Pulmonary Edema |
|  | Parenchymal Congestion |
|  | Alveolar Hemorrhage |
|  | Peribronchial Inflammation |
|  | Perivascular Inflammation |
|  | Interstitial Inflammation |
| **Liver Injury** | Ischemic Necrosis |
|  | Parenchymal Congestion |
|  | Hepatocellular Injury |
|  | Periportal Inflammation |
|  | Vacuolar Degeneration |
| **Renal Injury** | Congestion |
|  | Glomerular Necrosis |
|  | Tubular Necrosis |
| **Cardiac Injury** | Parenchymal Congestion |
|  | Necrosis |
|  | Inflammation |
| Scores for each criterion are given as 0, none; 1, mild; 2, moderate; 3, severe. At least three microscopic areas were examined to score each specimen. | |
