# Supplementary material for: Nuclear fragile X mental retardation-interacting protein 1-mediated ribophagy protects T lymphocytes against apoptosis in sepsis
Source: Burns Trauma. 2023 Feb 28;11:tkac055. doi: 10.1093/burnst/tkac055 (PMC9976742; doi:10.1093/burnst/tkac055)
Supplement: Supplementary_data_3_tkac055 [file supplementary_data_3_tkac055.docx]

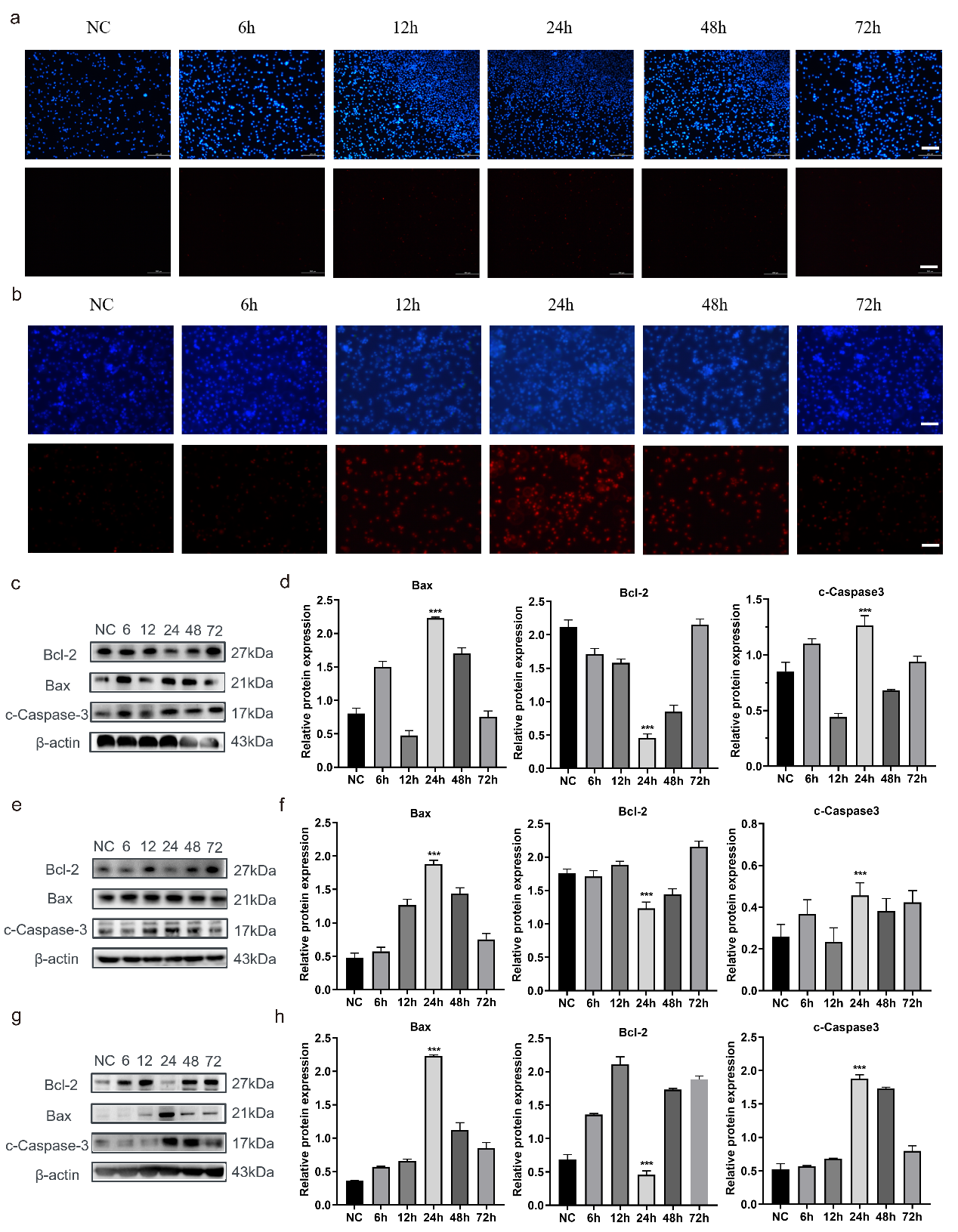


Supplementary data 3

**Supplementary data 3.** Apoptosis of T cells in the setting of sepsis.

(**a, b**) Apoptosis of splenic CD4^+^ T lymphocytes and *Jurkat* cells *in vitro* tested by TUNEL apoptosis assay. After LPS stimulation for 6, 12, 24, 48, and 72 h, the apoptosis showed a trend of an initial increase and then decrease, peaking at 24 h (Scar bar: 200 μm). (**c, d**) Apoptosis of splenic CD4^+^ T lymphocytes *in vitro* tested by WB. After LPS stimulation for 6, 12, 24, 48, and 72 h, the expression levels of Bax and c-Caspase-3 in splenic CD4^+^ T lymphocytes showed a trend of an initial increase and then decrease, peaking at 24 h. However, Bcl-2 expression showed a trend of an initial decrease and then increase, with the lowest value at 24 h. (**e, f**) Apoptosis of *Jurkat* cells *in vitro* tested by WB. (**g, h**) Apoptosis of splenic CD4^+^ T lymphocytes *in vivo* tested by WB. After CLP operation for 6, 12, 24, 48, and 72 h, the expression levels of Bax, c-Caspase-3, and Bcl-2 in splenic CD4^+^ T lymphocytes revealed a similar trend as *in vitro*, and the differences were most significant at 24 h. One-way ANOVA was applied to testify the statistical significance. Data were expressed as means ± SEM. **P* < 0.05, ***P* < 0.01, ****P* < 0.001. *NC* normal control group, *WB* western blot, *LPS* lipopolysaccharide, *CLP* cecal ligation and puncture, *ANOVA* analysis of variance, *SEM* standard error of mean.
