# Supplementary figures and images for: Nuclear fragile X mental retardation-interacting protein 1-mediated ribophagy protects T lymphocytes against apoptosis in sepsis
Source: Burns Trauma. 2023 Feb 28;11:tkac055. doi: 10.1093/burnst/tkac055 (PMC9976742; doi:10.1093/burnst/tkac055)

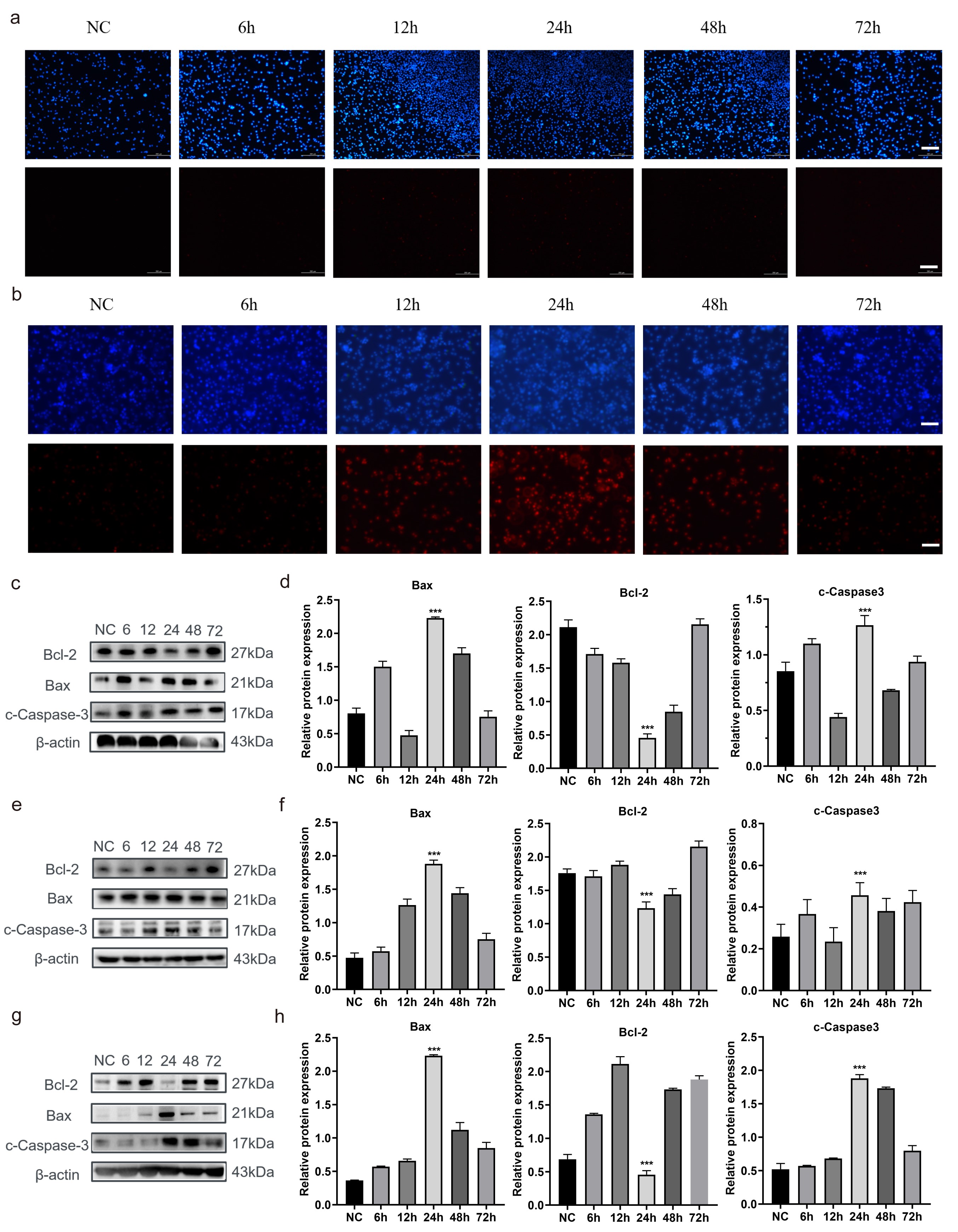

Supplement: Figure_S3_tkac055 [file figure_s3_tkac055.jpeg]
